# Editorial: Inter and intra-individual synchronization studies: a comprehensive review of the past 30 years

**DOI:** 10.3389/fpsyg.2023.1256987

**Published:** 2023-07-31

**Authors:** Michela Balconi

**Affiliations:** ^1^International Research Center for Cognitive Applied Neuroscience, Università Cattolica del Sacro Cuore, Milano, Italy; ^2^Research Unit in Affective and Social Neuroscience, Department of Psychology, Università Cattolica del Sacro Cuore, Milano, Italy

**Keywords:** EEG, neural synchronization, intra-individual, inter-individual, functional connectivity

Organic synthesis of the main basic and applied studies on the topic of intra- and interpersonal synchronization has allowed us to highlight how the methodology of intersubjective analysis is extremely useful for different sectors, from clinical to experimental, to applied neuroscience (Balconi et al., 2017b).

Clinical domains are among the main and most fervent applications of interpersonal synchronization studies, where the social neuroscience approach tests the own inner paradigm of analysis of interpersonal synchronization and attunement through a direct reference to contexts of care and assistance of psychological distress. According to Saul et al., the principle of two-person neuroscience is a relevant context of the application of the inter-individual synchronization approach, and it highlights the interpersonal aspect of social interactions by observing behavior and brain activity from both constituents of the interaction, rather than analyzing on an individual level or observing a social situation from an individual perspective. Therefore, this inter-individual approach could be a promising direction for the assessment and intervention of social distress, such as anxiety disorder (Balconi et al., 2019). The authors propose a novel paradigm that integrates two-person neuroscience in a neurofeedback protocol. Neurofeedback and interbrain synchrony were discussed to highlight their capacities for their relationship with social anxiety disorder and their relevance as a branch of two-person neuroscience.

The ability to use the “instrument” of synchronization as a method of multilevel analysis of human interactions in therapeutic contexts is well-highlighted by the experimental report by Tourunen et al., which focused on both patient–therapist pairs and married couples or colleagues, with significant innovative and, in some respects, counterintuitive results. In this study, the relationship between sympathetic nervous system synchrony, movement synchrony, and speech production in couple therapy was studied. The different roles and relationships in couple therapy were associated with the extent to which synchrony modalities were linked with each other, and relevant differences were found in correspondence to the type of relationship: in the relationship between patients and therapists, synchrony in arousal levels and movement “walked hand in hand”, whereas, in spouse or colleague dyads, they were not directly linked.

Similarly, linguistic synchrony plays a central role in the conditions in which the word becomes a vehicle of interpersonal harmony in interactions. In this case, the “food for thought” comes from Tay and Qiu's study, which focused on linguistic synchrony used as variables, to derive a global synchrony measure per dyad, and finally, qualitative analysis derived from each dyad: all aimed at providing examples of possible applications to different therapeutic matrices and models, such as psychoanalysis, cognitive behavioral, and humanistic therapy. The resulting synchrony measures reflect the general approach of these therapy types, also revealing that synchrony is contextually co-constructed.

But even more specific evidence about the widespread use of the social neuroscience and interpersonal synchronization approaches in the clinical setting comes from considerations about the association between synchronous interactions and the underlying mechanisms of prefrontal deficits in conditions such as autism spectrum disorder (ASD). Furthermore, the evidence of functional neuroanatomy confirms how much this approach and methodology of analysis constitute a valid tool today, even potentially with diagnostic application and implications, as highlighted by the contribution of Chan et al.. In this regard, abnormal interhemispheric and interhemispheric prefrontal hypoconnectivity were found to be related to deficits in executive processes that are essential for reading comprehension in ASD.

As a powerful biomarker of social interactions, their intrinsic quality and the role that synchronization can have as a “social” regulator of interpersonal dynamics, one of the most significant conditions in which to test this specific paradigm of social neuroscience is that of joint action, defined as synergistic actions between two or more inter-agents (Balconi et al., 2017a). An example among all is the joint action of a choir aimed at creating a single and inseparable “product” of performance. Elucidating the coordination dynamics, Delius and Müller considered this synergic performance with respect to the concept of a superordinate system, or superorganism, based on the principles of “self-organization” and “circular causality”. The authors confirmed that choral singing is a “dynamic process requiring tight interpersonal action coordination that is characterized by coupled physiological systems and specific network topology dynamics”. In this exact meaning, it represents a potent biomarker for social interaction.

Even more exciting is the possibility of using interpersonal synchronization as a scientific tool capable of disambiguating models, confirming their scientific validity, or verifying their stability in the comparison between social dynamics and the cognitive value of the models themselves. This was performed in Koshino et al.'s study, which discussed the default mode network (DMN), considering the question of whether it exhibits increased activation during the processing of social and personal information but shows deactivation during working memory (WM) tasks. In close association with models of a neuroanatomical nature, such as the frontal-parietal network (FPN), the authors discussed promising empirical results if the FPN and DMN showed coactivation during a specific process (task preparation) while the DMN exhibited deactivation during a different condition (task execution) in working memory tasks.

The broader and more open research perspectives of the interpersonal synchronization paradigm emerge regarding issues affecting subjective and intersubjective wellbeing, as in the case of considering the relationship between nature and distress response. Imperatori et al. have obtained promising results, through the use of electroencephalography (EEG) exploring the restorative cognitive/emotional effects of nature. By investigating the association between exposure to nature and EEG functional connectivity in the distress network, they suggested that experiencing natural environments is associated with brain functional dynamics linked to emotional restorative processes.

What better opportunity to be able to apply the research paradigm not only to clinical, therapeutic, and interpersonal relationships in everyday contexts but also to cognitive and social neuroscience.

## Author contributions

The author confirms being the sole contributor of this work and has approved it for publication.
